# Total knee arthroplasty using NexGen LPS-flex® improves clinical outcomes without early loosening: minimum 6-year follow-up results

**DOI:** 10.1186/s13018-016-0419-5

**Published:** 2016-07-21

**Authors:** Yoon Sang Jeon, Joong Sup Shin, Jae Ho Jung, Myung Ku Kim

**Affiliations:** Department of Orthopaedic Surgery, Inha University Hospital, 7-206, 3-Ga Sinheung-dong, Jung-gu, Incheon, 400-711 South Korea

**Keywords:** NexGen LPS-flex, Total knee arthroplasty, Aseptic loosening, High-flexion TKA

## Abstract

**Background:**

The authors analyzed clinical and radiological 6-year follow-up results after total knee arthroplasty (TKA) with NexGen LPS-flex® and implant survivorship.

**Methods:**

The medical records of 80 patients that underwent 122 TKAs using NexGen LPS-flex® from February 2005 to November 2008 and followed up for at least 6 years were reviewed. The Internal Knee Documentation Committee (IKDC) subjective form, Western Ontario and McMaster Universities Arthritis Index (WOMAC), and Knee Injury and Osteoarthritis Outcome Score (KOOS) scores and preoperative and postoperative ranges of motion (ROMs) were recorded. Radiological assessments were performed by simple radiography preoperatively, immediately postoperatively, and at the final follow-up.

**Results:**

At the last follow-up visits, average ROM improved from 115.0° (80°–135°) to 131.76° (80°–150°), average IKDC subjective score from 30.54 (13–48) to 53.53 (31–80), average WOMAC score from 59.81 (35–90) to 15.98 (1–47), and average KOOS score from 75.33 (38–115) to 115.0 (52–174). The clinical results of 66 knees that had >130° of postoperative flexion and 56 knees that had <130 of postoperative flexion were compared. Radiolucent lines were found in 7 knees in those with a flexion angle of >130° and in 6 knees in those with a flexion angle of <130°, but the lines did not progress and meaningful loosening was not observed. Similarly, the occurrences of radiolucent lines in those with a flexion angle of >130° or <130° were not significantly different (*p* > 0.05).

**Conclusions:**

TKA with NexGen LPS-flex® showed satisfactory clinical improvements, including high flexion, and no early loosening was found at 6-year follow-up visits.

## Background

Increased numbers of total knee arthroplasty (TKA) cases have resulted in many publications of clinical and radiological results, which usually have been favorable [1–3]. Improvements in knee function as well as pain relief can be of practical help after surgery. In particular, it is important that range of motion (ROM) be secured to achieve functional improvement of the knee. Generally, postoperative ROM has been reported to be between 110° and 120°, which is smaller than of the normal knee [4, 5]. However, this extent of ROM does not limit patients’ daily activities, but deep knee flexion is required in young and active individuals in Asian countries.

Much effort has been expended in address requirement in Asia, and this has resulted in improved surgical procedures and more advanced prosthesis designs, such as high-flexion total knee prostheses. The LPS-flex® system is a prosthesis that enables high flexion by extending the posterior condyle by 2 mm, thus increasing the contact area during flexion. Early outcome results were divergent and showed LPS-flex® had the same or greater flexion angle extents than conventional implants. Furthermore, when cases of early loosening after arthroplasty were reported, the clinical benefits of high-flexion total knee prostheses were questioned [6–10].

Mid-term results of TKA using high-flexion total knee prosthesis with clinically sufficient verification were in demand. We retrospectively analyzed and assessed (with a follow-up of least 6 years) mid-term results after TKA was conducted with the NexGen LPS-flex® high-flexion total knee prosthesis (Zimmer, Warsaw, IN, USA).

## Methods

### Study population

We reviewed the medical records of 80 patients that underwent 122 TKAs using NexGen LPS-flex® from February 2005 to November 2008 with a follow-up of at least 6 years. Mean study subject age was 66 (43–86) years at time of surgery, and average follow-up duration was 80.9 (72–132) months. The protocol of this study was approved by the Inha University Hospital Institutional Review Board (approval number: INHAUH 2016-01-016). Indications for surgery were as follows: degenerative arthritis in 111 knees, rheumatoid arthritis in 4 knees, traumatic arthritis in 3 knees, and spontaneous osteonecrosis of the knee in 4 knees.

### Surgical techniques and postoperative management

All surgeries were performed by the corresponding author. Prophylactic antibiotics were administered 1 h before surgery. An anterior midline skin incision was made and capsular incision undertaken using a medial parapatellar approach in all cases. Bone resections were performed using an extramedullary alignment guide system for tibiae and an intramedullary alignment guide system for femurs. Anterior and posterior cruciate ligaments were excised in all knees. Distal femurs were resected first and a valgus angle of 5°, 6°, or 7° was chosen based on differences between mechanical and anatomical axes derived from low extremity long view radiographs. Posterior femoral osteophytes were removed as much as possible, and femoral, tibial, and patellar prosthesis components were fixed with cement. Patellar resurfacing was performed in 31 of the 122 knees because of the presence of a broad cartilage defect of ICRS grade 3 or 4 on the patellar articular surface.

On the first or second postoperative day, the drainage hemovac was removed and continuous passive motion (CPM) was started. At the same time, the patients began active knee motion exercises by walking with crutches or a walker. At 2 weeks after surgery, stitches were removed and patients were discharged with crutches or a walker.

### Clinical assessment

All patients were clinically followed, and simple radiographs were taken at 3, 6, and 12 months and then annually thereafter in an outpatient clinic. Clinical parameters including Knee Injury and Osteoarthritis Outcome Score (KOOS) and Internal Knee Documentation Committee (IKDC) subjective form, Western Ontario and McMaster Universities Arthritis Index (WOMAC) scores, and preoperative and postoperative ranges of motion (ROMs) were recorded. We compared the postoperative ROMs of patients with a preoperative ROM of <120° with those of patients with a preoperative ROM of >120° and rates of achieving high knee flexion in these two groups. In addition, we also compared clinical and radiological outcomes of patients with a postoperative ROM of <130° with those of patients with a postoperative ROM of >130 at the final follow-up.

### Radiological assessment

Radiological assessments were done at the same times as clinical assessments using antero-posterior radiography taken in a standing position and lateral view using the radiological evaluation system of the Knee Society. Valgus angles of femoral components (*α*) and varus angles of tibial components (*β*) were measured on AP knee radiographs, and flexion angles of femoral components (*γ*) and posterior slopes of tibial components (*δ*) were measured on the lateral knee radiographs. A radiolucent line (RLL) was defined as any RLL of ≥1 mm. RLLs were sorted as physiological and pathological lines. A non-progressive 1- to 2-mm-thick RLL was defined as a physiological RLL. Also, a physiological RLL was generally surrounded by sclerotic margin and not considered loosening. But a pathological RLL was greater than a 2-mm-thick RLL and poorly defined progressive. It was related with aseptic implant loosening [11].

### Statistical analysis

The statistical analysis was performed using a *t* test and Wilcoxon’s signed rank test in SPSS ver. 22.0. Statistical significance was accepted for *p* values of <0.05.

## Results

### Clinical results

In all study subjects, average ROM increased from 115.0° (80°–135°) preoperatively to 131.76° (80°–150°) at the last follow-up, an average increase of 16° (*p* < 0.05). More than 95 % achieved maximum flexion ROM within 6 months, and in the remaining 5 %, flexion ROM gradually increased. Average IKDC subjective score increased from 30.54 (13–48) to 53.53 (31–80) at the last follow-up, average WOMAC score from 59.81 (35–90) to 15.98 (1–47), and average KOOS score from 75.33 (38–115) to 115.0 (52–174), and all increases were significant (*p* < 0.05) (Table 1).

**Table 1:** Clinical results

	Preoperative	Final follow-up	*p* value
Range of motion (°)	115.0°	131.76°	0.021
IKDC subjective	30.54	53.53	0.043
WOMAC	59.81	15.98	0.032
KOOS	75.33	115.0	0.001

We compared 60 knees with a preoperative ROM of <120° and 62 knees with a preoperative ROM of >120°. The average ROM in the <120° group increased significantly from 108.2° (80°–110°) preoperatively to 128.6° (80°–140°) at the last follow-up (*p* < 0.05), whereas average ROM in the >120° group decreased from 135.2° (120°–140°) to 133.8° (117°–140°). The rate of high knee flexion (>130°) postoperatively was 46.6 % in the <120° group (28 of 60 knees) and 56.5 % in the >120° group (39 of 62 knees), and these rates were significantly different (*p* < 0.05) (Table 2).

**Table 2:** Postoperative ranges of motion of cases in the < and >120° preoperative ROM groups

	Postoperative flexion group membership: number (%)	
	<130°	≥130°
<120° group	32 (53.4 %)	28 (46.6 %)
>120° group	23 (43.5 %)	39 (56.5 %)

Values are presented as numbers

In addition, we assigned 56 knees with <130° of postoperative flexion at the last follow-up to group 1 and 66 knees with >130° of postoperative flexion to group 2 and compared clinical results. In groups 1 and 2, average IKDC subjective scores were 50.30 and 56.27, respectively; average KOOS scores were 111.24 and 118.19, respectively; and average WOMAC scores were 15.62 and 16.28, respectively. IKDC and KOOS scores were higher in group 2 patients that achieved high knee flexion, but this difference was not significant (Table 3).

**Table 3:** Clinical results of cases in group 1 and group 2

	Group 1	Group 2	*p* value
IKDC subjective	50.30	56.27	0.500
WOMAC	15.62	16.28	0.515
KOOS	111.24	118.19	0.195

Group 1: <130° of flexion at the last follow-up, group 2: ≥130° of flexion at the last follow-up

### Radiological results

In all study subjects, average tibiofemoral angle was 4.78° varus preoperatively and 7.67° valgus at the final follow-up, which indicated that malalignments have been corrected (*p* < 0.05) (Table 4). Positional changes of components were analyzed using simple radiographs taken directly after surgery and at the last follow-ups. The mean valgus angle of femoral components (*α*) was 97.02° directly after surgery and 97.16° at the last follow-ups, and the mean varus angle of tibial components (*β*) was 90.35° directly after surgery and 89.74° at the last follow-ups. Mean flexion angles of femoral components (*γ*) directly after surgery and at the last follow-ups were 3.87° and 3.60°, respectively, and mean posterior slopes of tibial components (*δ*) directly after surgery and at the last follow-ups were 86.6° and 86.86° respectively. These changes in mean *α*, *β*, *γ*, and *δ* angles were not statistically significant (*p* > 0.05) (Table 5).

**Table 4:** The tibiofemoral angle in all patients

	Preoperative	Final follow-up	*p* value
Tibiofemoral angle	Varus 4.78°	Valgus 7.67°	0.032

**Table 5:** Radiological results in all patients

	Immediate postoperative	Final follow-up	*p* value
*α* (°)	97.02°	97.16°	0.726
*β* (°)	90.35°	89.74°	0.524
*γ* (°)	3.87°	3.60°	0.091
*δ* (°)	86.60°	86.83°	0.617

**Fig. 1:**
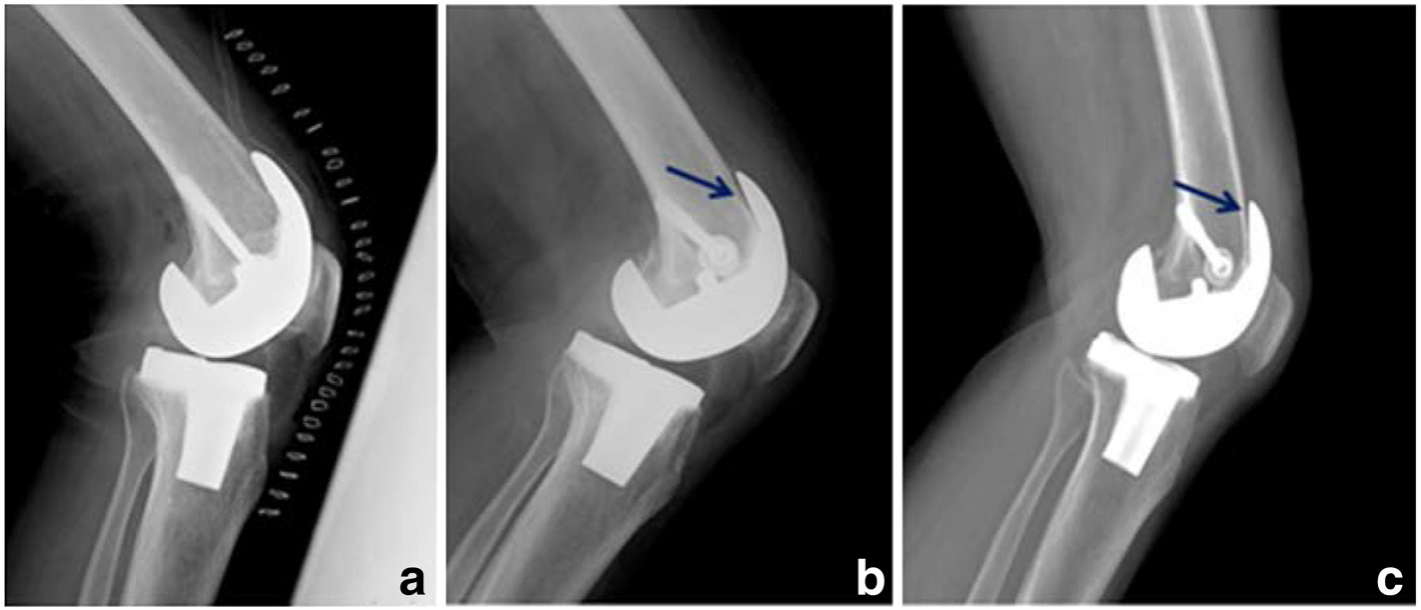
Left lateral knee radiograph showing a radiolucent line at the anterior surface of femoral component (*arrow*). **a** Immediately after surgery. **b** Postoperative 3 years. **c** Postoperative 5 years

In lateral radiographs, 7 of the 66 cases in group 2 (>130°) had an RLL and 6 of 56 cases in group 1 (<130°) had an RLL; however, all RLLs were less than 2 mm and did not progress. In serial radiographs, no patients complained of any symptom (Fig. 1). Eleven cases that had an RLL were confirmed at 6 months after the operation, and each 1 case in both group 1 and group 2 was indentified at 12 months after the operation. All 13 RLLs were observed at the femoral component (11 cases at the anterior surface and 2 cases at the posterior surface). No meaningful component displacement was observed, and neither aseptic loosening nor early failure (as indicated by a RLL) was encountered. No significant difference was observed between groups 1 and 2 in terms of RLL incidence or aseptic loosening (*p* > 0.05) (Table 6).

**Table 6:** Radiological results of the group 1 and the group 2

	Group 1	Group 2	*p* value
Radiolucent line	6	7	0.322
Aseptic loosening	0	0	>0.05

Group 1: <130° of flexion at the last follow-up, group 2: ≥130° flexion at the last follow-up

Values are presented as numbers

### Complications

There were 10 deep-vein thrombosis cases (an incidence of 12.5 %), and of these there were three cases of pulmonary embolism (3.75 %) and one case of cerebral infarction (1.25 %). However, all were discovered early and treated successfully without severe sequelae. In two cases (1.6 %), deep infection requiring revision surgery was diagnosed. In one of these cases, a patient with diabetes as an underlying disease complained of pain and heat sensation 2 years after surgery. Arthrocentesis was performed and methicillin-resistant staphylococcus aureus (MRSA) was cultured. PROSTALAC was inserted and revision surgery was performed 6 weeks after diagnosis. A favorable outcome without recurrence over 60 months of follow-up was achieved. In the other case, the patient complained of pain, heat sensation, and limitation of movement 3 years after surgery. Arthrocentesis was conducted under the suspicion of postoperative infection, and *E. coli* was identified. After revision TKRA, there were no evidence of recurrence over 48 months of follow-up, and ROM was recovered to 100°. Two postoperative herpes zoster infection cases occurred but without evidence of aseptic loosening.

## Discussion

LPS-flex® is a high-flexion total knee prosthesis that was designed to allow greater flexion angles than conventional LPS after TKA. Moon et al. reported that average ROM increased from 107.3° pre-operation to 122° at the final follow-up [7], whereas Kim et al. and Nutton et al. found no difference versus conventional LPS [10, 12].

Concerns have been voiced regarding early loosening because high-flexion total knee prostheses can weaken femoral component bone support by additional 2-mm bone resection from the posterior femur. And hyperflexion would cause to expose the component to overwhelming stress. Early loosening has been subject of considerable discussion because it has been reported that early aseptic loosening could be expected for high-flexion total knee prostheses. Han et al. reported that after a mean follow-up of 32 months early loosening of femoral components occurred in 38 % cases and that 21 % of these cases required revision surgery [13]. Cho et al. reported the presence of RLLs in 13.8 % of cases and found that the incidence rate was higher in cases with a larger postoperative ROM [14]. Choi et al. concluded early loosening was closely and positively related to compression force exerted on the posterior condylar when knee is flexed if the posterior part of the femoral component protrudes excessively and suggested this loosening was accelerated by the lever action of the femoral posterior condylar [15].

However, recent mid-term and long-term follow-up studies on LPS flex reported no early loosening. In a study that compared LPS with mobile and fixed types of LPS flex with a mean follow-up of 5 years, no difference was found between LPS flex and LPS in terms of survival rate [16]. Furthermore, Kim et al. failed to find any clinical difference between LPS flex and conventional TKA after a long-term follow-up and reported favorable outcomes and a 10-year survival rate of 99 % [17]. Rhee et al. also reported that high-flexion TKA yielded satisfactory clinical and radiological outcomes with a 99.2 % implant survival rate at 5 years postoperatively [18].

A recent study by Lee et al. with a 5-year follow-up reported a 99 % survival rate for LPS flex and no difference between the survival rates of cases that achieved high knee flexion and those that did not [11]. In the present study, no early loosening was found after a mean follow-up of 6 years. An RLL was observed in seven cases in group 2 (knee flexion of >130°) and in six cases in group 1. However, meaningful loosening was not found and no intergroup differences in clinical outcomes were evident. Furthermore, rates of RLL and aseptic loosening were no different in groups 1 and 2 (*p* > 0.05). In our study, complicated infection occurred in 2 of 122 knees, an infection rate of 1.6 %.

**Fig. 2:**
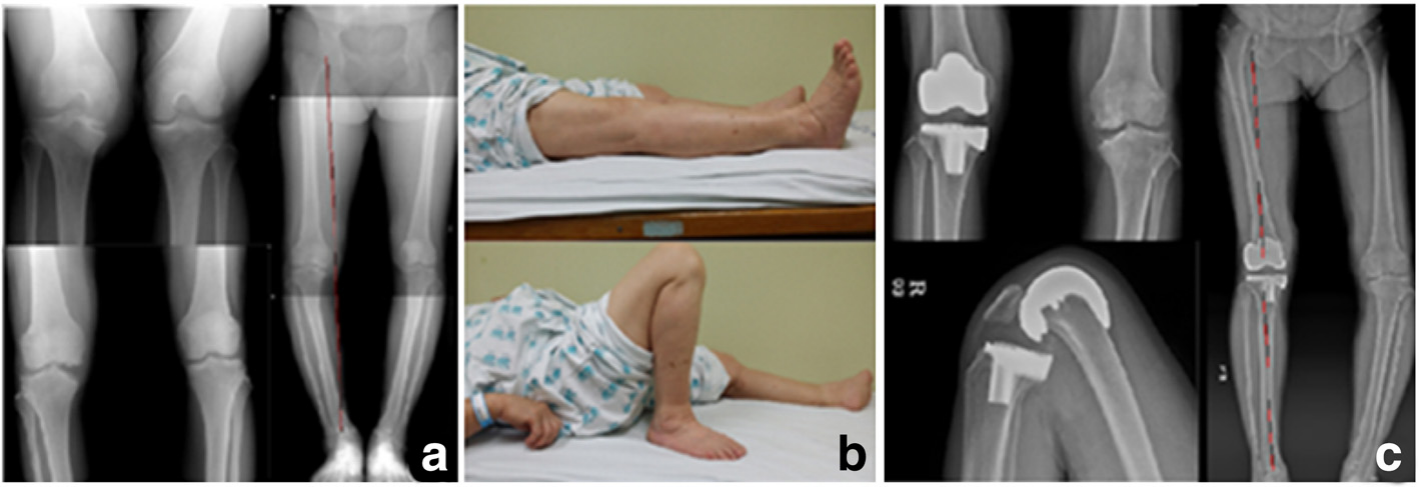
A 62-year-old female with right knee osteonecrosis underwent TKA using NexGen LPS-flex®. **a** Preoperative knee standing PA 45Flex, AP, low ex. long view. **b** The patient could perform ROM exercise fully (0°–150°) and comfortably at 92 months follow-up (final F/U). **c** Final follow-up knee AP, lateral full flexion and low ex. long views showing no loosening or wear and improved varus deformity

**Fig. 3:**
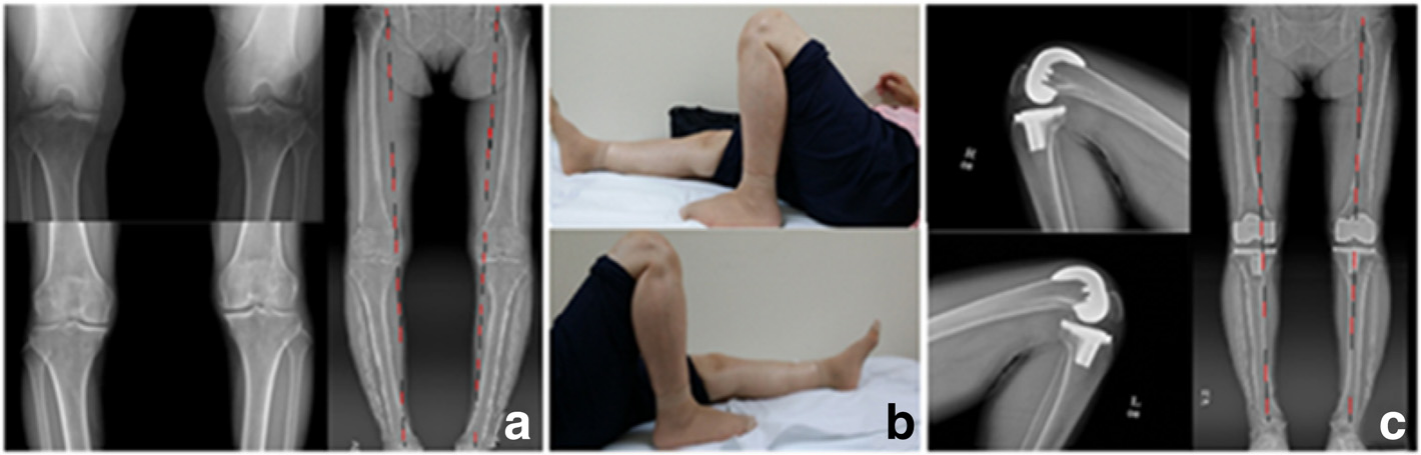
A 66-year-old female with both knee osteoarthritis underwent TKA using NexGen LPS-flex®. **a** Preoperative knee standing PA 45Flex, AP, low ex. long view. **b** The patient could perform ROM exercise fully (Rt. 0°–145°, Lt. 0°–140°) and comfortably at 80 months follow-up (final F/U). **c** Final follow-up both knee lateral full flexion and low ex. long views showing no loosening or wear and improved varus deformity

This study on the mid-term results of LPS flex documents significant ROM and clinical outcome improvements after surgery with no case of early loosening (Figs. 2 and 3). Nevertheless, we cannot conclude that these outcomes are better than those of conventional TKA. In order to demonstrate the superiority of high-flexion total knee prostheses, we recommend a long-term follow-up, comparative study be conducted.

## Conclusions

TKA with NexGen LPS-flex® produced satisfactory clinical improvements, which included high flexion with no early loosening, after a mean follow-up of 6 years. Furthermore, we failed to find any significant difference between the RLL occurrence rates of knees that achieved high flexion and those that did not.

## Abbreviations

CPM, continuous passive motion; ICRS, international cartilage repair society; IKDC, Internal Knee Documentation Committee; KOOS, Knee Injury and Osteoarthritis Outcome Score; MRSA, methicillin-resistant staphylococcus aureus; RLL, radiolucent line; ROM, range of motion; TKA, total knee arthroplasty; WOMAC, Western Ontario and McMaster Universities Arthritis Index
